# Men, Women, and Bastards

**Published:** 1918-03-16

**Authors:** 


### MEN, WOMEN, AND BASTARDS.

### Some Pressing Questions of Great Importance.

On this subject a correspondence has been carried on in the *Times* with respect to the treatment that is and the treatment that ought to be accorded to the girl who has, in bygone phrase, "fallen"—in current phrase, to the mother or the expectant mother of an illegitimate child.

Mrs. H. B. Irving writes a harrowing account of the pale, trembling girl, within a month of her confinement, being bullied and baited by a board of brutal Guardians, who told her they hoped she would suffer severely for her wrongdoing, abused her mother for not casting her off, and expressed the belief, nay, the hope, that the man who was father to the coming child would take an early opportunity of abandoning her. Of course, this was very hard, and very brutal, and very unjust, if it all took place as Mrs. Irving describes it; but she declines to specify the Guardians who behaved thus, and we have not heard their account, which might perhaps put a different complexion on the matter.

There is a good deal of rather mawkish sentimentality afloat with respect to bastard children and unmarried mothers, and it is very necessary that our minds should be cleared of cant with respect to it. In the first place, it must never be forgotten that marriage is the foundation of society, and that female chastity is of enormous importance to the community and the nation. A nation in which female chastity is not valued is foredoomed to destruction, and no small share in the downfall of the Greek republics, and even of Imperial Rome itself, is to be attributed to the loosening of the marriage tie and to the indifference with which feminine chastity came to be regarded. A girl who has "fallen," who has become pregnant without being married, who has lost her chastity, may in some cases be an object of sympathy, and may properly be treated with consideration and even tenderness, but to slop over with maudlin sentimentality and to treat her as a martyr, and even, as is now becoming the fashion, as a meritorious person to be glorified and adulated, to be made the spoiled pet of idle women, to take the place of the Pekinese spaniel or the toy Pomeranian, is disgusting and demoralising; yet there is a decided tendency at the present time to treat such girls in this way. On the other hand, to hound such girls into the streets, to blame their mothers for not turning them out of doors, to treat them as utter pariahs, outcast from all decent society, is atrocious. There is a middle way.

It is not possible to lay down any rule that will apply to every case without inflicting cruel injustice upon some. At one end there is the girl who is deliberately seduced by premeditated design, whose mind is first corrupted by inducing her to read demoralising literature, stimulating the passions and flouting religion and morality, glorifying vice, and treating decency with contempt and ridicule. Then opportunity is made; she is entertained at dinner and plied with wine; a late train is designedly missed; even a mock marriage may be resorted to; and at length the design of the seducer is accomplished. Such cases are described in works of fiction; in real life they are excessively rare. It would not do to say they never happen, but they are excessively rare.

The more ordinary case is that time, place, and opportunity being favourable, two young people deeply in love, and closely associated, are carried away by their passions, and it would puzzle the Recording Angel himself to apportion more fault to the one than to the other. No doubt the man, as the most ardent, is the first to transgress, but no doubt the woman fails to meet the transgression with that resentment that would at once put a stop to it.

But there is a third case that is by no means rare, and that is scarcely recognised as generally as it should be. Men are ardent, it is true, but the vast majority of men, especially of young men, are also extremely modest in the presence of a virtuous woman, or a woman whom they believe to be virtuous; and there is a very large class of cases in which not the man, but the woman, is the seducer. Many a woman deliberately lays herself out to entrap a man for what she can get out of him. If a single man, to entrap him into marriage; if a married man, to blackmail him by threats of exposure. Such cases are very frequent indeed, and the artful women who engineer them are just the women to pose as victims of designing man, and to win the sympathy of silly sentimental women, as well as of hard-headed business men who are unprepared for such depth of designing. It is for these women that the natural aversion to sexual immorality of the naturally virtuous woman proves so useful. Hard common sense, reinforced by instinctive aversion to vice, and unblinded by the male sensibility to personal good looks and attractiveness, sees through such women in a moment, and correctly relegates them to their proper class of "designing huzzies."

It is abundantly manifest that in the first case the man is wholly to blame, that his conduct is abominable, and that the woman deserves a great deal of sympathy. It is abundantly manifest that in the third case the woman alone is to blame, and that all our sympathy should be reserved for the man. As to the middle class, we must make allowance for the weakness of human nature, and remember that *tout comprendre, c'est tout pardonner*. It must be remembered also, however, that though cases do occur in which there is neither design nor premeditation by either party, yet there are very varying degrees of readiness to yield to a temptation that presents itself, and that this readiness may show itself on the part of the man or of the woman; so that, as aforesaid, there is no rule by which every case should be judged. Each must be judged on its own merits as far as the immorality of the transaction is concerned, and as

**MEN, WOMEN, AND BASTARDS—(*continued*).**

far as blame or sympathy is to be apportioned to either party, and much difficulty is often experienced in ascertaining the merits. Consider the following cases: A young woman in her early twenties falls deeply in love with a man, who returns her affection in full. He is called away to the war, and leaves her pregnant and near her confinement. The case falls into our second class. The girl is without friends. Her parents are dead, or probably this would not have happened. She is a Roman Catholic, and her priest adjures her that she has committed a deadly sin, and that her only chance of saving her immortal soul is never to see the man again, to go into a reformatory, to be parted from her baby as soon as it can be weaned, and to expiate her sin by lifelong mortification and repentance. However, a kind-hearted and sensible woman of the world heard of the case, visited the girl, provided her with medical attendance, baby-linen, and so forth, and put to her the crucial question, Do you love him? On receiving a fervent answer in the affirmative, the visitor said: " Then never part from him." The priest visited this Good Samaritan, and assured her with indignation that she was destroying all chance of salvation, not only for the wretched girl's soul, but for her own also. However, the lady said she was willing to take the risk; the priest shook the dust off his orthodox boots as he left the house; the soldier came home and married the girl; and whatever their fate may be in the next world, it is certain that in this the couple is much happier for following the advice of the lady rather than that of the priest.

In the second case the facts are very different, and are less certain. What is certain is that a very wealthy woman has a son whom she adores, and a confidential maid of attractive appearance, that the maid became pregnant by the son; that the mother turned the maid out of doors, and took the son more fondly to her heart than ever. How are we to apportion to the parties the blame in such a case as this? It is obvious that we cannot come to any decision until we know more of the facts. Does the case come in the first, the second, or the third class? If in the first, the mother is grossly to blame, and mother and son are of the same stock. If in the second, the mother has been wanting in humanity to the woman, and has shown gross partiality towards the man. If in the third, the mother has acted as any mother might be expected to act, and ought to act.

Again we say to our readers, Clear your minds of cant. Don't treat these women with indiscriminate and draconic severity. Don't treat them with indiscriminate and sloppy sentimentality. Suspend your judgment until you know the facts, having heard both sides of the matter. Remember that the preservation of a high standard of feminine chastity is of vital importance to the nation and to morality in general; but remember also that *tout comprendre c'est tout pardonner*. It is right that those who have erred should suffer. It is wrong to persecute even the erring. The punishment of the girl who has gone wrong must in any case be heavy. She has a child to support without the assistance of its father, without the glory of a family home of her own. This is the inevitable punishment of her fault. It is unnecessary to add to it. There is no harm in alleviating it. Condemn the girl in your mind if you please; but you need not add reproaches to your condemnation; and while you are condemning the girl, do not forget to measure out condemnation to the man if investigation shows that he deserves it. In most cases he does deserve it; and in most cases he escapes it. This is grossly unfair.

As long, however, as mankind is divided into two sexes, a time the end of which we cannot yet foresee, there will be injustice in this matter. Sex will display itself, even in the most even-minded. Women will always be more ready to condone the faults of men than those of women; men will always be more ready to condone the faults of women than of men. It would be a bad day for the nation and for society generally if the lapse from feminine virtue should be lightly regarded. To regard it, as it would seem some are inclined to regard it now, as rather praiseworthy than otherwise would be indeed calamitous.

